# Non-targeted Metabolomics Analysis Reveals Distinct Metabolic Profiles Between Positive and Negative Emotional Tears of Humans: A Preliminary Study

**DOI:** 10.7759/cureus.42985

**Published:** 2023-08-05

**Authors:** Hao Liang, Songye Wu, Duo Yang, Jianhua Huang, Xiaolei Yao, Jingbo Gong, Zhixing Qing, Lijuan Tao, Qinghua Peng

**Affiliations:** 1 Institute of Traditional Chinese Medicine Diagnostics, Hunan University of Chinese Medicine, Changsha, CHN; 2 Ophthalmology Department, Jili Hospital, Liuyang, CHN; 3 Institute of Herbs, Hunan University of Chinese Medicine, Changsha, CHN; 4 Ophthalmology Department, First Hospital of Hunan University of Chinese Medicine, Changsha, CHN; 5 Psychiatric Disease Clinical Research Center, Shanghai Changning Mental Health Center, Shanghai, CHN; 6 Hunan Key Laboratory of Traditional Chinese Veterinary Medicine, Hunan Agricultural University, Changsha, CHN; 7 Ophthalmology Department, Hunan Children's Hospital, Changsha, CHN

**Keywords:** tears, types of tears, metabolomics, emotional evaluation, emotional tears, metabolic profile

## Abstract

Background

Basal, reflex, and emotional tears differ in chemical components. It is not yet known whether chemical differences exist in tears of different emotions. We investigated the biochemical basis of emotional tears by performing non-targeted metabolomics analyses of positive and negative emotional tears of humans.

Methods

Samples of reflex, negative, and positive emotional tears were obtained from 12 healthy college participants (11 females and one male). Untargeted metabolomics was performed to identify metabolites in different types of tears. The differentially altered metabolites were screened and assessed using univariate and multivariate analyses.

Results

The orthogonal partial least squares discriminant analysis model showed that reflex, negative, and positive emotional tears were clearly separated. A total of 133 significantly differentially expressed metabolites of electrospray ionization source (ESI-) mode were identified between negative and positive emotional tears. The top 50 differentially expressed metabolites between negative and positive emotional tears were highly correlated. Pathway analysis revealed that secretion of negative emotional tears was associated with some synapses in the brain, regulation of a series of endocrine hormones, including the estrogen signaling pathway, and inflammation activities, while secretion of positive emotional tears was correlated with biotin and caffeine metabolism.

Conclusions

It is indicated that metabolic profiles of reflex, positive, and negative emotional tears of humans are distinct, and secretion of the tears involves distinct biological activities. Therefore, we present a chemical method for detecting human emotions, which may become a powerful tool for the diagnosis of mental diseases and the identification of fake tears.

## Introduction

Three types of tears are produced in humans: basal, reflex, and emotional [1]. Basal and reflex tearing serve the human eyes and their physiological functions are fully understood. Basal tear is the small quantity of tear produced to maintain a lacrimal film on the corneal surface. Reflex tear is a lacrimal flow produced in response to irritations, such as inflammation and foreign body invasion. However, the origin and mechanisms of emotional tearing are poorly studied. Emotional crying is a unique human behavior, resulting from cognitive and psychogenic brain processes [2]. Even though emotional tears also originate from the eyes, they do not serve the organ itself. Emotional tears are communicating signals; they express the need for help or empathic responses. This mysterious behavior has fascinated both scientists and laypeople, but research on this topic is still at the primary stage. Efforts should be aimed at revealing the neurophysiological and biochemical underpinnings of the behavior.

Human newborns exhibit vocal crying when they are separated from their mothers. This behavior is consistent across different species of mammals and birds and requires no previous learning. In this period, the shedding of visible tears is triggered by strong contractions of the orbicularis oculi muscles during the production of distress vocalizations, which stimulate the sensitive corneal sensory nerves [3]. Real emotional tearing appears later than reflex tearing, several months after birth [4]. It is reported that most children exhibit emotional tearing nearly in the sixth week of life while Vignat et al. found that emotional tearing occurs at about four months of age [2].

Tears evolve with increasing age to serve as emotional signals that convey complex information. In infants and children, physical pain and discomfort are important triggers of tears. However, adults and elderly people seldom cry because of physical conditions, and their weeping is usually associated with different emotional states [5]. As an essential additional feature of crying in humans, what is the role of emotional tears? Tearful crying results in tension reduction and has health benefits. The biochemical hypothesis suggests that in negative situations, the (endogenous) release of endorphins or oxytocin while crying is cathartic to the criers [6]. Adults cry in negative situations, such as losses, failures, and helplessness, as well as in positive situations, such as when witnessing the intensification of relationships, prosocial behaviors, and happiness [5].

Tears are composed of proteins, lipids, metabolites, and electrolytes. Chemical components of tears differ among basal, reflex, and emotional tears [7,8]. However, chemical variations in tears of different emotions are yet to be established. Tears of mice contain a chemosignal or pheromone [9]. Gelstein et al. reported that human tears also contain a similar chemosignal [10]. We hypothesized that different emotional tears (positive or negative emotions) contain distinct substances, which form the basis of chemosignals for tearing. Therefore, we investigated the biochemical basis of emotional tears via non-targeted metabolomics analysis of negative and positive emotional tears of humans.

This article was previously posted to the medRxiv preprint server on February 02, 2022 (https://doi.org/10.1101/2022.01.28.22270049).

## Materials and methods

Study population

Ethics Statement

This is a diagnostic accuracy test design study. The protocol for this study was approved by the Ethical Committee of Jili Hospital (No. 2021-04; date: May 27, 2021) and was conducted in accordance with the Declaration of Helsinki. The study is registered in the China Clinical Trial Registration Center (Registration No. ChiCTR2100047025, http://www.chictr.org.cn/showproj.aspx?proj=127637). Moreover, written informed consent was obtained from each participant.

Inclusion and Exclusion Criteria

The inclusion criteria for study participants were as follows: aged between 17 and 35 years; no physical diseases during the recent routine health check.

The exclusion criteria were as follows: refractive measurement of ≤ -6.0 D or > +5.0 D; catarrhal inflammation in the recent seven days due to upper respiratory tract infections and conjunctivitis among others; eye surgery within the recent six months; febrile illnesses within the recent seven days; a history of mental illness, such as depression, autism, and schizophrenia, or recent psychic trauma; long duration of insomnia (>one month); smokers (>10 cigarettes per day) and drinkers (drink every day).

All volunteers were subjected to ophthalmic examinations between June 1st and September 30th, 2021. The examination included visual acuity, refraction, slit-lamp, and tear film breakup time to confirm the healthy states of their ocular surfaces. Fifty healthy students (30 females and 20 males) from the Hunan University of Chinese Medicine met the inclusion criteria and were finally recruited into this study.

Study design and sample collection

Tear Types and Identification

Essential balm composition was daubed on the bags beneath the eyes to stimulate the production of reflex tears. Negative-themed (sadness, loss, betrayal, torture, persecution, and discrimination) movies were selected to help produce tears of negative emotions (distressed, upset, guilty, scared, nervous, and afraid emotions) while positive-themed (happiness, love, friendship, faith, and loyalty) movies were selected to help produce tears of positive emotions (excited, strong, enthusiastic, inspired, and determined).

After watching several of the selected movies, the research team voted for the movies that could induce negative or positive emotional tears as follows.

Negative: (1) The Chinese medical documentary *Life Matters* (S2EP01, https://www.youtube.com/watch?v=knJ3t-GeUyc). It is a story documenting the end stage of children with cancer in a hospital. (2) The movie Manchester by the Sea.

Positive: (1) The movie *The Pursuit of Happyness*. (2) The movie *Hachi: A Dog’s Tale* (2009).

Essential balm-induced reflex tears were labeled the C samples (control). Emotional tears were induced by watching different movies. After emotional tears had been successfully induced and collected, participants were immediately required to finish the Positive and Negative Affect Schedule (PANAS-SF), which is a scale consisting of different words that describe feelings and emotions [11]. The participants described emotions according to the scale at that moment to shed tears. If the positive affect score of PANAS-SF ranged from 10 to 50, the tear sample was labeled M (positive, induced by moving scenes), or else the sample was classified as S (negative, induced by sad scenes) when the negative affect score ranged from 10 to 50. To avoid individual variations, the three types of tears were all from the same participants.

Collection of Tears

Each participant was requested to watch a movie and collect tear samples in a private room between 16:00 and 21:00. Sleeping time of the participants during the past 24 hours should have exceeded six hours. The use of contact lenses within the past 24 hours was also forbidden. Before the sample collection process, the face was washed to clean any makeup on the eyelids, eyelash, and face.

**Figure 1:**
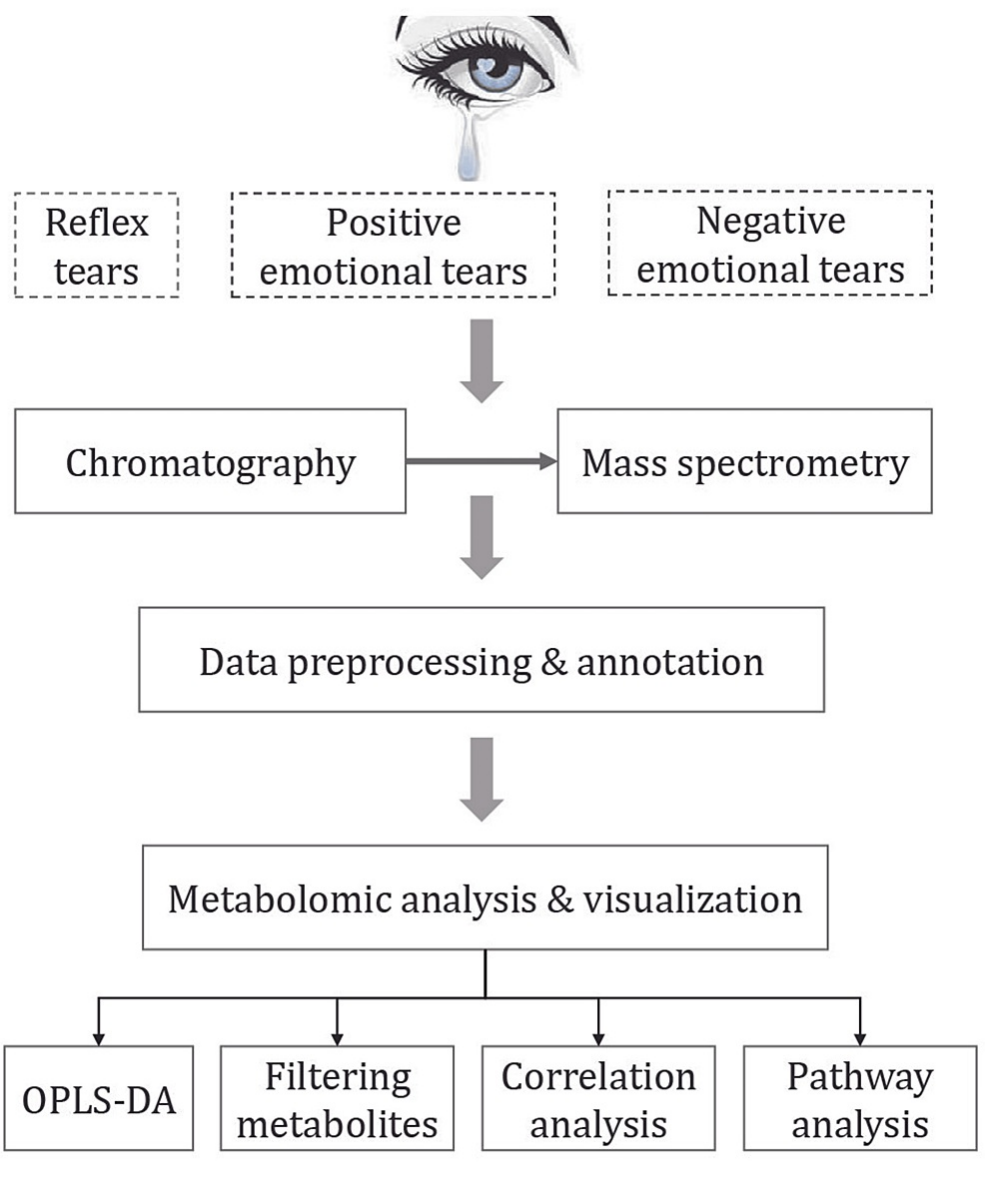
Workflow of tears collection and analysis. OPLS-DA: orthogonal partial least squares discriminant analysis.

The Schirmer tear test strips were used for collecting tears from both eyes. The Schirmer strip was bent at the preformed notch at 90° and placed into a conjunctival sac of the participant at the junction of the mid and temporal thirds of the lower lid when the first drop of tears flowed out of the eyelid. The volume of a 35 mm Schirmer strip is 35 μL. The length of the moistened area was measured using the millimeter scale on the strip. Three Schirmer strips (wetting of more than 25 mm for each strip) collecting each type of tear from every participant were placed in a 2.0 mL sterile cryogenic vial, frozen, and immediately stored in a -80°C refrigerator for metabolomic analyses. The collecting interval between different tear types for each participant was more than 24 hours. Finally, different tear types (reflex, positive, and negative emotional tears) were obtained from the 12 participants. The workflow for this study was as shown in Figure 1.

Metabolomics analyses

Sample Preparation

Samples were taken out from the -80°C refrigerator and thawed on ice. The redundant part was cut off to keep each of the Schirmer strips at 24 mm length. Then, 70% methanol-water internal standard extractant (300 μL) was mixed in the corresponding Eppendorf tube with sample number, vortexed for five minutes, sonicated in an ice water bath for 10 minutes, and allowed to stand still at -20°C for 30 minutes. The mixture was centrifuged (12,000 rpm, 4°C) for 10 minutes, and 200 μL of the supernatant was transferred to a new centrifugal tube. The supernatant was centrifuged (12,000 rpm, 4°C) for three minutes and 150 μL of the supernatant was subjected to liquid chromatography-tandem mass spectrometry (LC-MS/MS) analyses. Moreover, 20 μL of each tear sample from each group was pipetted into a centrifuge tube to prepare a quality control (QC) sample for LC-MS/MS analysis. The researchers who performed the metabolomics analyses were blinded to the types of samples.

Chromatography

Chromatographic separation was performed using a Shimadzu LC-20AD UPLC system (Shimadzu Scientific Instruments, Kyoto, Japan) equipped with a Waters ACQUITY UPLC HSS T3 C18 column (1.8 µm, 2.1 mm * 100 mm; Waters Corporation, New Delhi, India). The column temperature was set at 40°C while the flow rate was 0.40 mL/min. The gradient system consisted of 0.1% formic acid in ultrapure Water in mobile phase A and 0.1% formic acid in acetonitrile in mobile phase B (0 minutes, 5% B; 11 minutes, 90% B; 12 minutes, 90% B; 12.1 minutes, 5% B; 14 minutes, 5% B).

Mass Spectrometry

Tandem mass spectrometry (MS/MS) was performed using an AB Sciex TripleTOF® 6600 quadrupole time-of-flight (QTOF) mass analyzer (AB Sciex LLC, Framingham, MA). Mass calibration was conducted daily, as instructed by the manufacturer. An electrospray ionization (ESI) source was used for mass spectrometric detection in positive and negative ionization modes. The QC samples were used for MS/MS data acquisition and source parameters are as shown in Table S1.

Data analysis

Data Preprocessing and Annotation

The original data file obtained through LC-MS analysis was first converted into the mzML format using the ProteoWizard (Palo Alto, CA) software. Peak extraction, alignment, and retention time correction were performed using the XCMS program. Then, the support vector regression (SVR) method was used to correct the peak area [12]. Peaks with deletion rates > 50% were filtered in samples of each group. Then, the metabolic identification information was obtained by searching the self-built database of the lab, public databases (Metlin and Human Metabolome Database (HMDB)), as well as MetDNA.

Statistical Analysis and Visualization

Principal component analysis (PCA) was used to reduce the dimensionality of the multidimensional dataset. Orthogonal partial least squares discriminant analysis (OPLS-DA) was used to establish metabolite differences among the tear types (Q2 > 0.50). A validation plot was used to assess the validity of the OPLS-DA model by comparing 200 random permutations of the Y variable and goodness of fit (R2Y and Q2) [13]. The corresponding variable importance in the projection (VIP) values were calculated using the OPLS-DA model and the VIP value > 1 indicated significant differences. The T-test (p < 0.05) and fold change (FC; FC ≥ 2 or FC ≤ 0.5) were used to screen the differentially expressed metabolites. Pearson correlation and cluster analyses were conducted to determine the correlations between the differentially expressed metabolites. Further, Kyoto Encyclopedia of Genes and Genomes (KEGG) enrichment analyses were performed to identify the pathways that were associated with emotional tears. Statistical analyses and visualization were performed using the R software (version 4.1, The R Foundation, Vienna, Austria).

## Results

Demographic information

Thirty-six tear samples (12 samples for each tear type, i.e., reflex, positive, and negative emotion) were obtained from the 12 participants (11 females and one male). Ophthalmic examinations of the participants did not reveal abnormal outcomes (Table S2).

Metabolomic analysis

QC Samples Analysis

In ESI+ and ESI- modes, the total ion chromatogram (TIC) of tears in QC samples was as shown in Figure S1. The overlap of the TIC curves was high, exhibiting good stability of mass spectrometry (MS) for the same sample at different times. The PCA plot for all samples revealed that the QC samples were tightly clustered (Figure S2), indicating good analytical reproducibility of the current metabolomics study.

Metabolic Profiles of Different Tears Types

**Figure 2:**
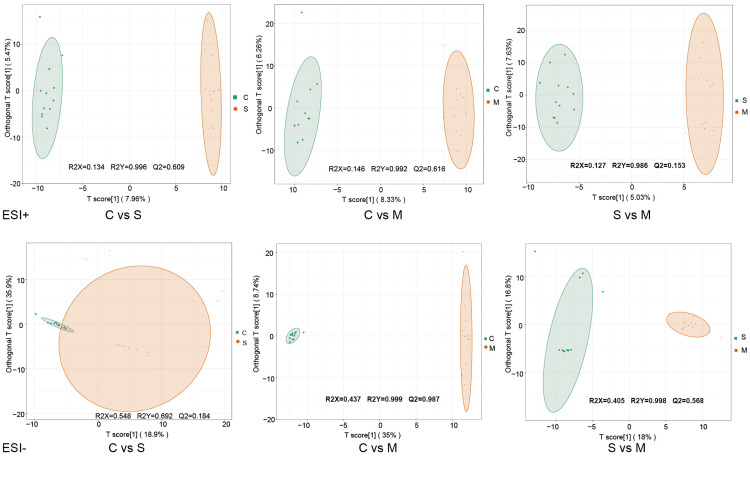
OPLS-DA score plots of multivariate statistical analysis between reflex tears, negative emotional tears, and positive emotional tears. C Group: reflex tears; S Group: negative emotional tears; M Group: positive emotional tears; OPLS-DA: orthogonal partial least squares discriminant analysis; ESI: electrospray ionization.

The OPLS-DA plot (Figure 2) revealed a remarkable separation between reflex (C), negative emotion (S), and positive emotion (M) tears. Specifically, the OPLS-DA score between C and S resulted in an R2Y = 0.996 and Q2 = 0.609 for ESI+ mode. The OPLS-DA score between C and M revealed R2Y = 0.992 and Q2 = 0.616 for the ESI+ mode, as well as R2Y = 0.999 and Q2 = 0.987 for the ESI- mode. The OPLS-DA score between S and M resulted in an R2Y = 0.952 and Q2 = 0.568 for ESI- mode. Moreover, validation analysis as presented in Figure S3 supports the reliability and good fitting of the OPLS-DA model in C vs. S and C vs. M of the ESI+ mode, as well as C vs. M and S vs. M of the ESI- mode, because the p-values for permuted R2Y and Q2 were all under 0.05.

**Figure 3:**
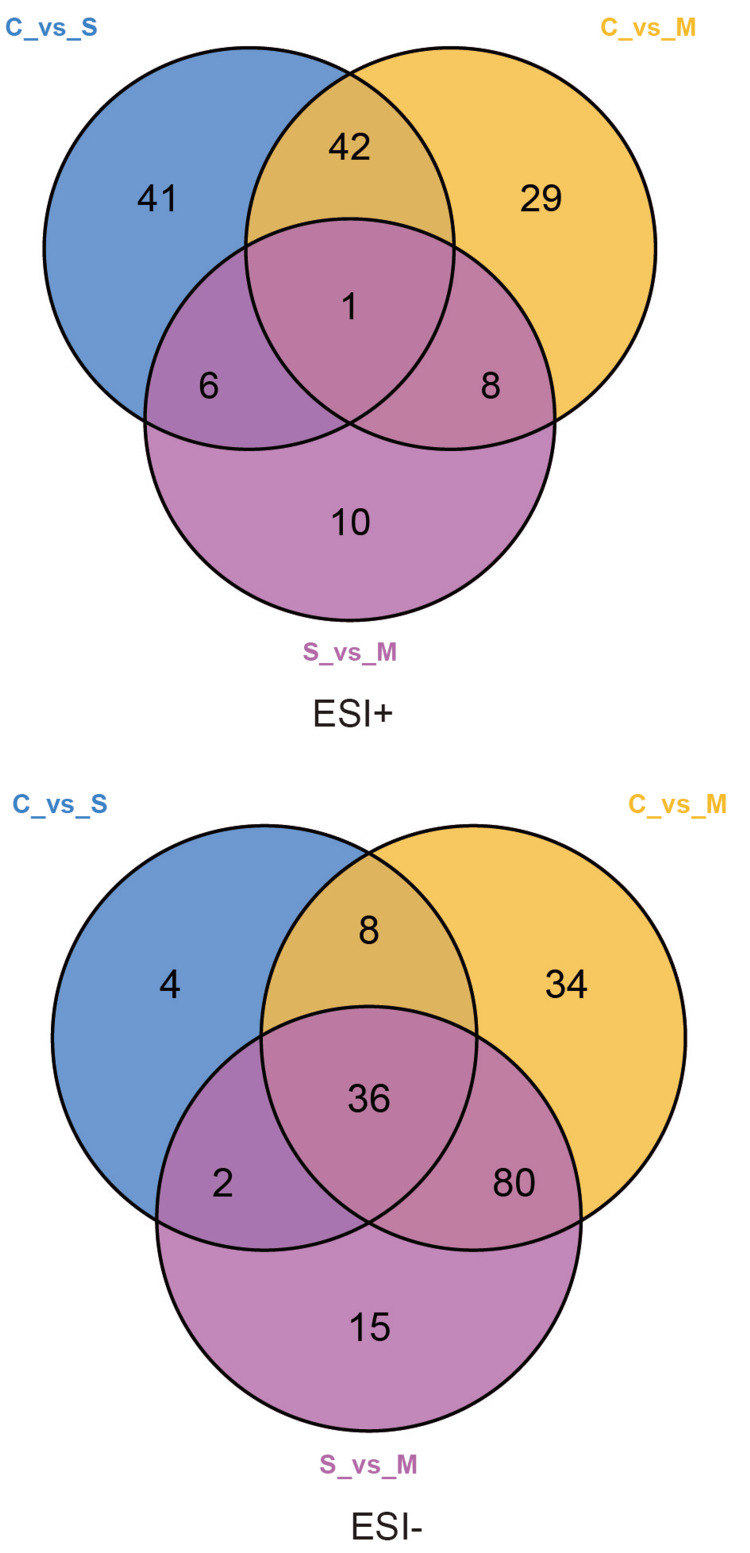
Venn plots of screened metabolites between reflex tears, negative emotional tears, and positive emotional tears. ESI: electrospray ionization.

The criteria for screening the differentially expressed metabolites were VIP value > 1, p-value < 0.05, and FC ≥ 2 or FC ≤ 0.5. There were 90 and 80 differentially expressed metabolites in C vs. S and C vs. M of the ESI+ mode, respectively. The top five differentially expressed metabolites (based on VIP value) between C and S were γ-dodecalactone, 24-epibrassinolide, PA(18:3(6Z,9Z,12Z)/16:0), 12-ketodeoxycholic acid, and 2-(2,6-dimethoxy-4-prop-2-enylphenoxy)-1-(3,4,5-trimethoxyphenyl) propan-1-ol. The top five differentially expressed metabolites between C and M were 2-phenylacetamide, (S)-cotinine N-oxide, γ-dodecalactone, lauric acid, and brinzolamide. Moreover, 158 and 133 differentially expressed metabolites were identified between C vs. M and S vs. M of ESI- modes, respectively. The top five differentially expressed metabolites between C and M of the ESI- mode were alprazolam, dethiobiotin, (-)-threo-Iso(homo)2-citrate, 1,3-diacetoxy-4,6,12-tetradecatriene-8,10-diyne, and maculine. The top five differentially expressed metabolites between S and M were 1,3-diacetoxy-4,6,12-tetradecatriene-8,10-diyne, indole-3-acetic acid, free fatty acids (FFA) (18:0), nopaline, and N6-acetyl-L-lysine. These metabolites are displayed in a volcano (Figure S4) and Venn (Figure 3) plots. A total of 133 significantly differentially expressed metabolites were identified between negative and positive emotional tears in the ESI- mode (Table S3).

Correlation Analysis of Differentially Expressed Metabolites

**Figure 4:**
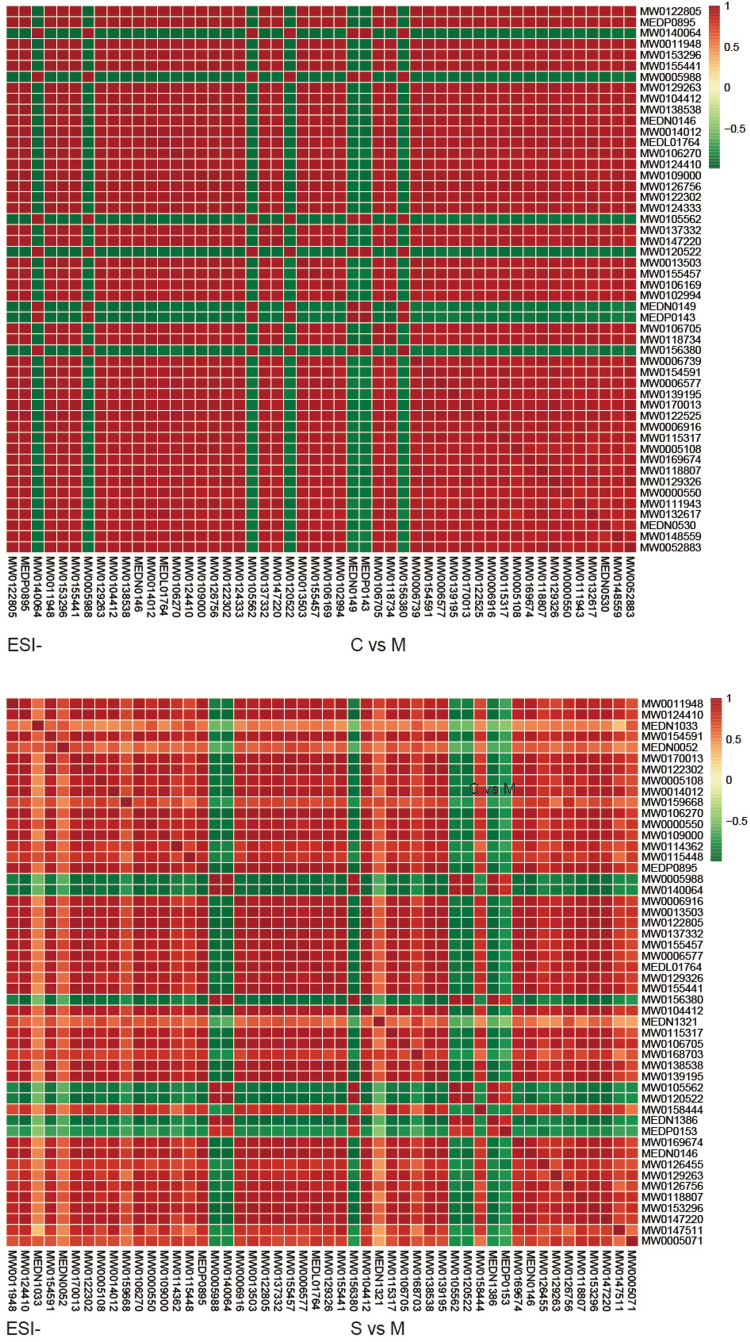
Correlation plots of the top 50 differentially expressed metabolites between reflex tears, negative emotional tears, and positive emotional tears. C Group: reflex tears; S Group: negative emotional tears; M Group: positive emotional tears; ESI: electrospray ionization.

The relationships between the differentially expressed metabolites and the different classifications of tears were assessed via cluster and Pearson correlation analyses. Heatmaps of cluster analysis show relative intensity distributions and relationships of the metabolites. Metabolites in different groups were divided using color. The downregulated and upregulated metabolites are closely clustered (Figure S5). Pearson correlation analysis was also performed to assess the relations among the top 50 differentially expressed metabolites (VIP value) in each mode. Heatmaps of correlations showed that metabolites in C vs. M and S vs. M of the ESI- mode were highly correlated (Figure 4).

The Enriched Metabolic Pathways

**Figure 5:**
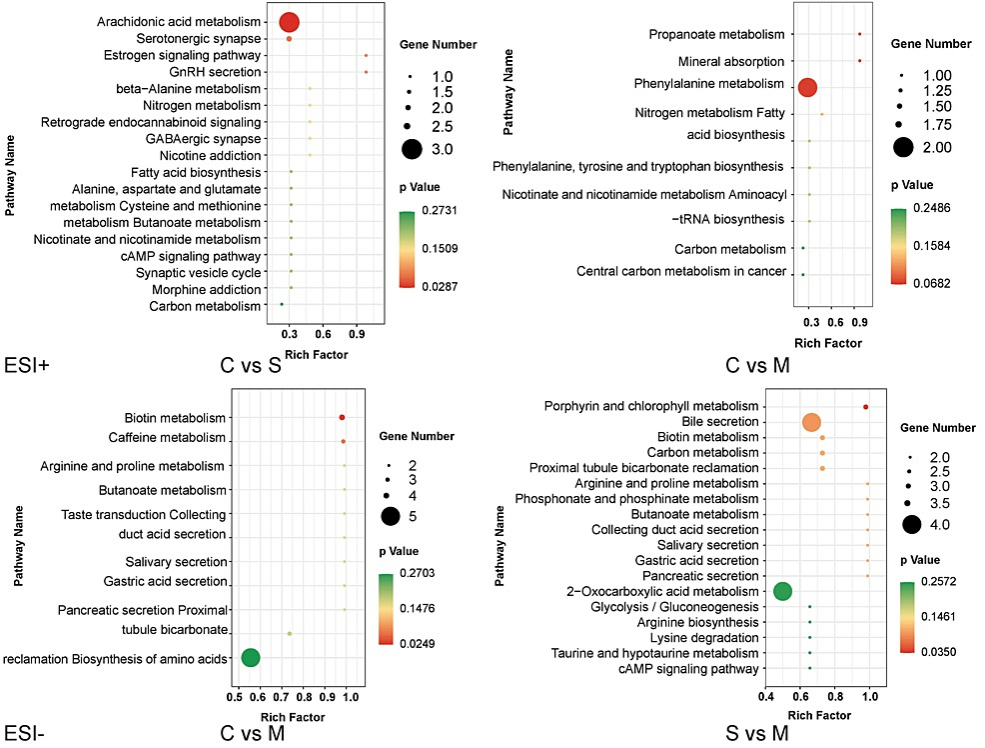
KEGG enrichment scatter plots of the identified differentially expressed metabolites between reflex tears, negative emotional tears, and positive emotional tears. C Group: reflex tears; S Group: negative emotional tears; M Group: positive emotional tears; KEGG: Kyoto Encyclopedia of Genes and Genomes; ESI: electrospray ionization; GnRH: gonadotropin-releasing hormone.

The ClusterProfiler package was used to analyze the relationships between the differentially expressed metabolites after which all the annotated metabolites were mapped into biochemical pathways for mechanistic interpretations. The KEGG enrichment scatter plot was used to show the significantly related pathways (p < 0.30 and Rich factor > 0.20). The most enriched pathways in C vs. S (Figure 5) were arachidonic acid metabolism, serotonergic synapse, estrogen signaling pathway, gonadotropin-releasing hormone (GnRH) secretion, and beta-alanine secretion and metabolism. The most enriched pathways in C vs. M (Figure 5) were propanoate metabolism, mineral absorption, phenylalanine metabolism, and nitrogen metabolism of the ESI+ mode, as well as biotin metabolism and caffeine metabolism of the ESI- mode. The most enriched pathways of S vs. M (Figure 5) were porphyrin & chlorophyll metabolism, bile secretion, biotin metabolism, arginine & proline metabolism, as well as phosphonate & phosphinate metabolism.

Re-evaluation of Screened Metabolites

The second-level MS spectra for the top five differentially expressed metabolites between the groups were re-evaluated since some of the predicted metabolites were not endogenous from human beings. Then, we predicted other substances using different methods as references for future work (Table 1).

**Table 1:** Re-evaluation of the screened metabolites. Endo: endogenous human metabolite; RT: retention time; Score: prediction score from second-level mass spectrometry spectrums.

ID	Group	Formula	RT	Name	Score	Endo
MW0010466	C vs. S +	C23H30O7	614.976	2-(2,6-dimethoxy-4-prop-2-enylphenoxy)-1-(3,4,5-trimethoxyphenyl)propan-1-ol	0.6549	No
		C23H29F3O6	614.976	5-trans fluprostenol	0.6573	Yes
		C17H24N4O3	614.976	Lys-Trp-OH	0.6067	Yes
MW0127045	C vs. S +	C12H22O2	11.1875	γ-dodecalactone	0.6256	No
		C12H22O2	11.1875	δ-dodecalactone	0.6061	No
		C12H22O2	11.1875	cis-5-dodecenoic acid	0.5820	Yes
MW0153296	C vs. M -	C13H9NO4	3.1094	Maculine	0.5779	No
		C3H7NO2S	3.1094	D-cysteine	0.5405	Yes
MW0122805	C vs. M -	C17H13ClN4	5.1059	Alprazolam	0.7589	No
		C15H16O7	5.1059	4-[3-(3,4-dihydroxyphenyl)-2-hydroxypropyl]benzene-1,2,3,5-tetrol	0.7258	Yes

## Discussion

The untargeted metabolomics analysis based on the LC-MS/MS technique in the present study showed that different tears types revealed distinct metabolomic profiles. Visual separation was explicit from OPLS-DA score plots and Q2 values in C vs. S and C vs. M of ESI+ mode, as well as C vs. M and S vs. M of ESI- mode are all greater than 0.50, which is acceptable for classification in human metabolomic studies [14,15]. Specifically, 133 differentially expressed metabolites were identified between positive and negative emotional tears in ESI- mode. Correlation analysis revealed significant positive or negative correlations between the top 50 differentially expressed metabolites based on the VIP score. Moreover, some metabolites, such as indole-3-acetic acid (MW0124410) and free fatty acids 18:0 (FFA 18:0, MEDN1033) were only up-regulated in positive emotional tears while some metabolites were up-regulated in both positive and negative emotional tears. Various metabolites, such as 1,3-diacetoxy-4,6,12-tetradecatriene-8,10-diyne (MW0011948) and nopaline (MW0154591), were abundant in positive emotional tears. Some metabolites, such as (-) threo-iso(homo)2citrate (MW0140064) and aloe-emodin (MW0005988), were down-regulated in positive emotional tears. These results indicate that metabolite abundances significantly differed between positive and negative emotional tears. Therefore, the filtered metabolites are potential biomarkers for the detection of real emotions in humans.

Tears are made up of water, electrolytes, proteins, lipids, and mucins that form layers on the surface of the eyes [16]. The compositions vary significantly in different tear types (basal, reflex, and emotional). Humans are the only mammals known to produce tears in response to emotional states, such as joy or grief. Secretion of emotional tears may also serve a biological function by excreting stress-inducing hormones built up through times of emotional distress [17] and a form of social signaling, such as eliciting help and support from those around you. Emotional tears contain higher protein content. Therefore, they are more viscous, sticky to the skin, and take longer to roll down the face [18]. On the contrary, reflex tears are more dilute to help wash out any irritants to your eyes from foreign particles or vapors. They contain more antimicrobial compounds, such as lysozyme and defensin peptides, to prevent infections [19]. We found that the levels of some anti-bacterial metabolites, such as 24-epibrassinolide [20], were higher in reflex tears than in the other tear types. Studies on tears are still at the preliminary stage, and a lot of unknown components are yet to be identified. Even though we have proved that metabolites from different emotional tears are not identical, there is a need to confirm the biomarkers for diagnosing different emotions from tears.

The KEGG enrichment analysis of C vs. S revealed that secretion of negative emotional tears is associated with the regulation of some synapses in the brain, including serotonergic synapse and GABAergic synapse, and regulation of a series of endocrine hormones, such as the estrogen signaling pathway and GnRH secretion. Importantly, it also involves arachidonic acid metabolism, which plays a key role in inflammation [21,22]. Therefore, negative emotional crying may induce fluctuations in sex hormones and inflammatory activities.

On the other hand, KEGG enrichment analysis of C vs. M revealed that shedding of positive emotional tears is closely associated with biotin and caffeine metabolism. Brain areas demanding higher levels of biotin metabolism include the centers of auditory and visual activities [23], implying that biotin plays a pivotal role in activating carboxylases and the development of neurological diseases [24]. As the most commonly used stimulant drug for brain activities, caffeine improves vigilance and cognition [25], and the engendering positive emotional tears may be comparable to caffeine stimulation.

Positive emotional tears shedding is also associated with arginine and proline metabolism. The panel of metabolites associated with depression in animal models and patients has reported that levels of arginine, proline, taurine, glycine, and alanine are higher in depressed than in healthy individuals [26,27]. Microbial functions and metabolites, including proline, converging in glutamate/GABA metabolism are linked to depression. For instance, proline supplementation in mice exacerbated depression, along with microbial translocation [28]. Positive emotional tears production is also related to a series of external secretion pathways in the present study, such as salivary secretion, gastric acid secretion, and pancreatic secretion. Brain electrophysiological activities and salivary secretion can improve negative emotions and reduce amino acid (arginine, proline, histidine, and taurine) concentrations in saliva [25]. In summary, the secretion of either positive or negative emotional tears involves different biological activities.

Psycho-emotional weeping demonstrates mental responses to the environment and elicits sympathy as well as social support from observers [29,30]. Fake tears or “crocodile tears” are an insincere tearing display that can sometimes be used to manipulate and deceive; for instance, a disguise to get off charges in court [31]. They originate from ancient Greeks who had an anecdote in which crocodiles would pretend to weep while luring their prey in. “Crocodile tears” are typically related to simulated tears of celebrities and politicians, and conveying fabricated remorse during criminal court proceedings [31,32]. Detection of emotional deception is crucial in the social credit system, such as in commercial negotiations, charitable causes, and court trials. This study provides a convenient and promising way to expose emotional deception via chemical approaches.

The main limitation of this study is the small sample size. Although 50 participants were recruited, only 12 (24%) successfully completed the emotional tear collection process, probably because an adult person crying in front of a collector is rather embarrassing and difficult. Thus, because of the limited sample size, the diagnostic performance of the metabolites cannot be evaluated by the receiver operating characteristic (ROC) curve and area under the curve (AUC). However, the results are satisfactory because, in contrast to other metabolomics studies, tear samples of different types in this study were from the same 12 subjects, without individual variations. Another limitation was confounding factors, such as gender differences with regard to crying, whereby women are definitely better at it than men; thus, only one man (1/20) finished the emotional tears collection process. Furthermore, there are no tear metabolite databases, and the identification of metabolites as well as KEGG enrichment in this study was based on traditional algorithms and metabolite databases of human blood and urine. Therefore, the results of metabolite detection and metabolic pathways analysis in this study are inconsistent with reality. A tear omics database should be established for biomarker exploration and enrichment analyses.

## Conclusions

We analyzed the metabolites in 36 tears samples from 12 participants to investigate the metabolic characteristics between reflex, positive, and negative emotional tears using the non-targeted LC-MS/MS metabolomics approach. There were marked variations in metabolites among the three tear types, suggesting that tear metabolomics has great potential for human emotional detection. Moreover, the secretion of positive and negative emotional tears is associated with different biological activities, including biotin metabolism, arginine, and proline metabolism, among others. Therefore, our findings form a basis for the development of chemical methods for detecting human emotions, and for the diagnosis of mental diseases and the identification of fake tears.

## Figures and Tables

**Figure 6:**
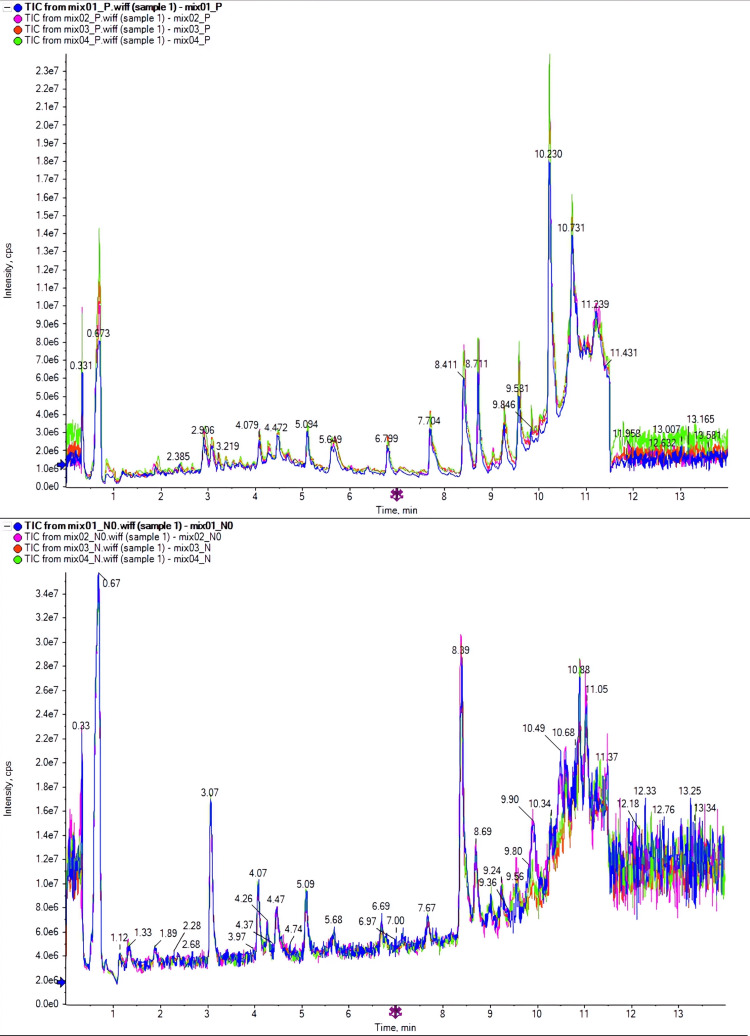
Total ion chromatogram (TIC) of tears in the quality control (QC) samples.

**Figure 7:**
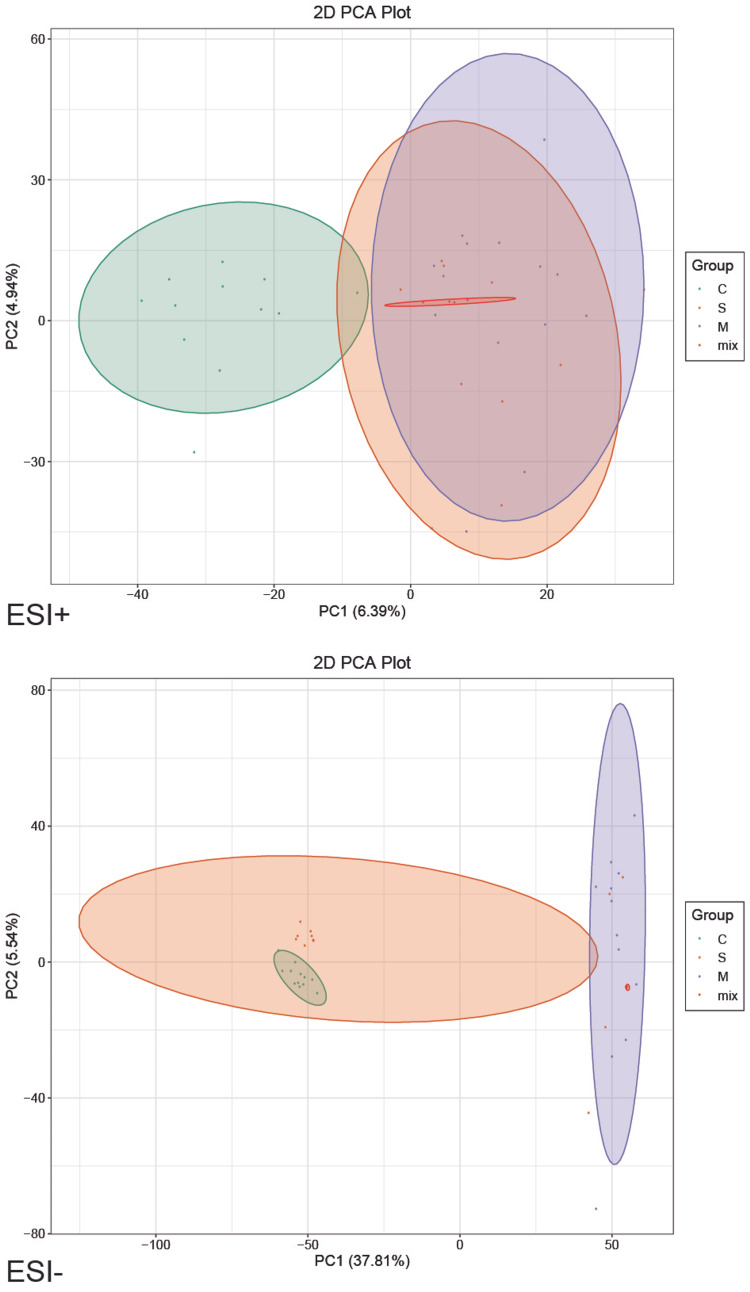
Principal component analysis (PCA) of whole samples. C Group: reflex tears; S Group: negative emotional tears; M Group: positive emotional tears; ESI: electrospray ionization.

**Figure 8:**
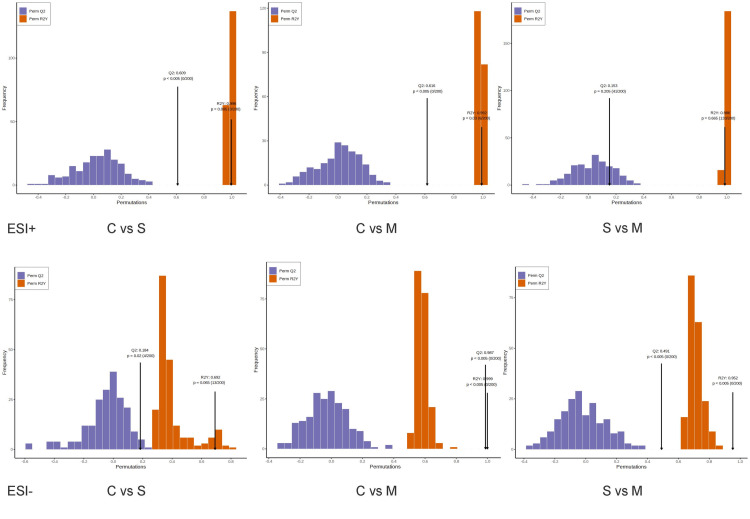
Validation analysis by comparing the goodness-of-fit (R2 and Q2) of OPLS-DA models with the goodness-of-fit of 200 Y-permutated models. C Group: reflex tears; S Group: negative emotional tears; M Group: positive emotional tears; OPLS-DA: orthogonal partial least squares discriminant analysis; ESI: electrospray ionization.

**Figure 9:**
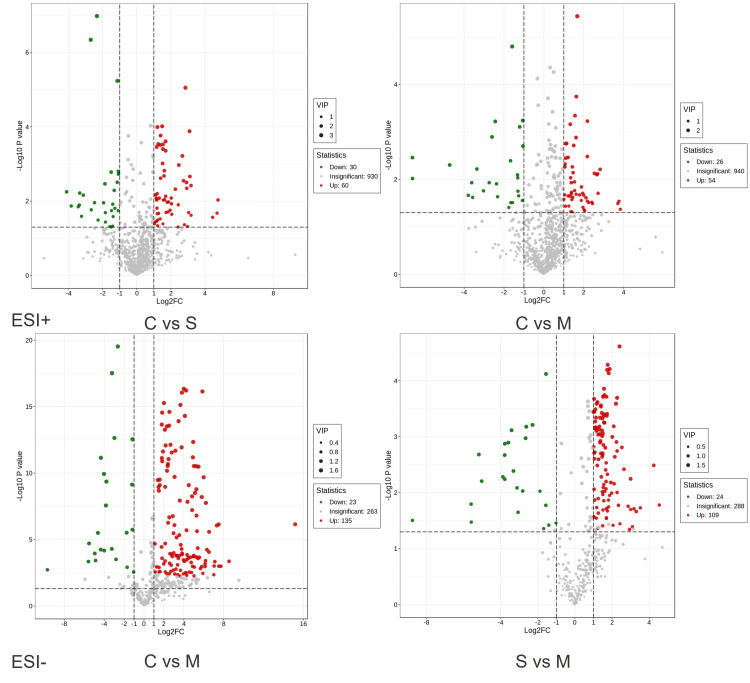
Volcano plots of differentially expressed metabolites. C Group: reflex tears; S Group: negative emotional tears; M Group: positive emotional tears; ESI: electrospray ionization.

**Figure 10:**
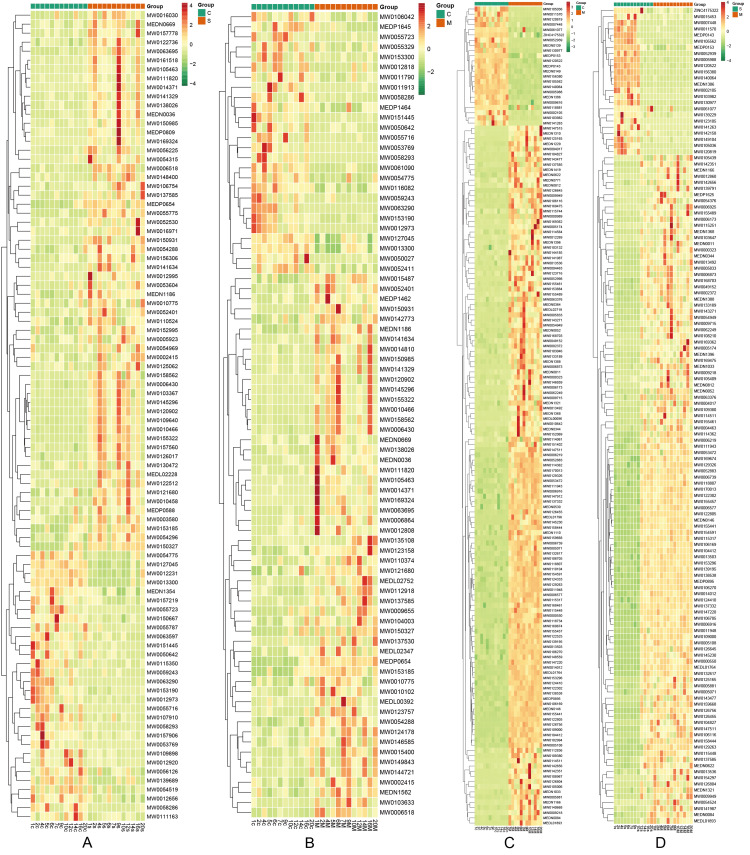
Heatmaps of cluster analysis of the overall differentially expressed metabolites. A: ESI+ C vs. S; B: ESI+ C vs. M; C: ESI- C vs. M; D: ESI- S vs. M; C Group: reflex tears; S Group: negative emotional tears; M Group: positive emotional tears; ESI: electrospray ionization.

## Appendices

The supplementary figures and tables can be downloaded from https://doi.org/10.6084/m9.figshare.21408768.

Figure S1

Figure S2

Figure S3

Figure S4

Figure S5

Table S1

**Table 2:** The source MS/MS parameters. MS/MS: tandem mass spectrometry.

Parameter	ESI+	ESI-
IonSpray Voltage	5,500	-4,500
Ion source gas 1	50	50
Declustering potential (DP)	60	-60
Collision energy spread	15	15
Ion release width	15	15
Curtain gas	35	35
Temperature	550	550
Ion source gas 2	60	60
Collision energy	30	-30
Ion release delay	30	30

Table S2

**Table 3:** Ocular surface examinations of participants. BUT: tear break-up time; L: left eye; R: right eye.

No.	Birth	Sex	BUT(L)-first	BUT(L)-avg.	BUT(R)-first	BUT(R)-avg.	Red-eye scan	Other
1	2001.9	Female	18.16	20.29	20.86	20.69	Negative	Eyestone
2	2002.2	Female	5.61	12.02	19.30	22.14	Negative	
4	2002.6	Female	5.68	15.73	8.03	15.00	Negative	
5	2002.5	Female	20.27	22.94	20.33	22.35	Negative	
6	2001.3	Female	6.75	17.34	3.63	10.60	Negative	Conjunctival pigmentation
7	2002.5	Female	10.90	16.70	4.69	12.36	Negative	
9	2001.12	Female	5.86	11.30	8.98	17.75	Negative	
10	2002.7	Female	22.94	23.40	7.07	13.13	Negative	
12	1986.12	Male	5.42	12.17	6.31	15.66	Negative	
14	1989.6	Female	7.91	10.23	11.28	14.53	Negative	
16	2000.7	Female	19.18	19.66	10.32	19.37	Negative	
20	1993.9	Female	7.33	12.71	19.69	21.35	Negative	

Table S3

**Table 4:** Significantly differentially expressed metabolites (top 10) of ESI- mode between negative and positive emotional tears. ESI: electrospray ionization; FFA: free fatty acids

Index	Compounds	Formula	Precursor (g/mol)	Mass (g/mol)	Score	RT (min)	MS_level	CAS_ID	HMDB_ID	cpd_ID	Metlin_ID	Pubchem.CID	Class.I	Class.II	Source	1s	2s	4s	5s	6s	7s	9s	10s	12s	14s	16s	20s	1M	2M	4M	5M	6M	7M	9M	10M	12M	14M	16M	20M	VIP	p-value	Fold_Change	Log2FC	Type
MW0011948	1,3-diacetoxy-4,6,12-tetradecatriene-8,10-diyne	C18H20O4	335.1034	300.1362	0.5077	4.0852	MS2-Insilico	29576-66-7	HMDB0030922	--	--	131751099	Lipids and lipid-like molecules	Fatty acyls	rp	4078.7	3781.91	4743.45	3366.38	3676.65	3323.08	3080.38	3.36	30517.07	28279.7	3319.95	26017.29	22900.88	27070.26	27122.4	22703.63	26853.0	29263.75	24935.77	27783.71	30617.02	29340.11	30117.96	28707.28	1.9083621044205163	0.00019274884831816985	2.8673415716828887	1.5197137756703256	up
MW0124410	Indole-3-acetic acid	C10H9NO2	174.055	175.0633	0.6363	4.0853	MS2-Search-DB	87-51-4	HMDB0000197	C00954	--	802	Organoheterocyclic compounds	Indoles and derivatives	rp	42326.27	36927.66	20.9	45723.61	8.67	37404.05	38556.47	51951.01	143231.71	130676.78	122531.05	161411.07	148706.98	146886.6	144230.73	145499.84	177103.72	134494.27	156837.65	143132.48	169676.01	156432.96	150456.97	154431.26	1.8026757658684305	0.0002575232676103954	2.254512575557102	1.172815557277837	up
MEDN1033	FFA (18:0)	C18H36O2	283.2563	284.2715	0.8577	11.4088	MS2-Search-Local	57-11-4	HMDB0000827	C01530	--	--	FA	FFA	rp	75470.79	94787.27	71648.32	60859.92	68215.24	19.21	124063.32	83906.84	56310.7	189641.99	86508.19	66825.87	103876.87	321012.73	556786.25	257220.01	527847.98	393178.25	497127.27	421123.94	6950.69	427442.09	715732.81	300150.61	1.7967367095449402	0.0002568037808020118	4.629096898663692	2.2107307621887657	up
MW0154591	Nopaline; D-nopaline; N2-(D-1,3-dicarboxypropyl)-L-arginine; N-[(1S)-4-carbamimidamido-1-carboxybutyl]-D-glutamic acid	C11H20N4O6	341.0919	304.1383	0.6495	6.02	MS2-MetDNA	22350-70-5	--	C01682	--	108012	Organic acids and derivatives	Carboxylic acids and derivatives	rp	514.51	6.19	9.28	8.89	6.96	722.66	1004.85	1895.76	7977.08	6715.72	5904.04	8301.61	5285.09	6279.56	8355.69	6967.58	8055.53	10815.43	8912.82	7807.61	9338.57	6819.43	10186.63	9318.21	1.783167004139699	0.0001389048904649927	2.9679292841471474	1.5694567177587069	up
MEDN0052	N6-acetyl-L-lysine	C8H16N2O3	223.0941	188.116	0.5834	6.26	MS2-MetDNA	692-04-6	HMDB0000206	C02727	--	92832	Amino acid and its metabolomics	Amino acid derivatives	rp	370.15	415.44	13.34	503.75	358.88	519.94	538.95	383.32	3924.21	147.34	72.21	4119.9	532.95	6409.84	5273.44	4606.9	5452.68	8780.54	4596.74	4694.33	4181.44	4824.13	5401.36	196.33	1.7758084775539775	0.00020214900681613798	4.834046042069317	2.27323121417688	up
MW0170013	Vulpinic acid	C19H14O5	321.0636	322.0841	0.5953	5.1052	MS2-Search-DB	521-52-8	--	--	44073	54690323	--	--	rp	11743.48	14.05	9841.35	10165.96	10337.24	11344.66	12214.86	9116.26	49591.63	47083.03	42764.51	54355.07	47200.12	40685.86	42500.84	55636.01	59274.58	64657.99	55603.51	54153.84	60810.3	50371.66	48219.09	44471.63	1.7725980410170212	0.00024157323803183721	2.321854838979924	1.2152777785643052	up
MW0122302	6-Quinoxalinecarbonitrile, 1,2,3,4-tetrahydro-7-nitro-2,3-dioxo-	C9H4N4O4	266.9898	232.0233	0.6222	5.6002	MS2-Search-DB	115066-14-3	--	C13668	--	3721046	Organoheterocyclic compounds	Diazanaphthalenes	rp	492.64	60.22	72.64	50.43	108.39	438.73	1330.73	13.93	6451.32	5268.16	6716.86	7020.77	4862.41	5738.94	6947.75	7693.81	7703.85	7783.95	6323.42	7800.87	8762.17	6843.76	6399.51	7521.55	1.771034976231105	0.00018132456196628678	3.0109734870732447	1.5902300045633035	up
MW0005108	4-hydroxymandelonitrile	C8H7NO2	165.0716	149.0477	0.5356	4.2807	MS2-Search-DB	-	--	C00650	--	166768	Benzenoids	Phenols	rp	65.88	3.15	5.44	3.15	155.96	145.45	310.98	354.21	2766.3	3718.76	1928.86	2186.74	3941.9	4244.89	3094.09	3320.11	2908.38	2933.55	3590.01	2587.11	3596.83	2226.74	2264.56	4114.48	1.7678814114042791	6.405247396614681e-05	3.3338814998522963	1.7372028257959218	up
MW0014012	3-dehydroepiandrosterone sulfate	C19H28O5S	404.1346	368.1657	0.8469	7.6549	MS2-MetDNA	651-48-9	--	C04555	34483	12594	Lipids and lipid-like molecules	Steroids and steroid derivatives	rp	3602.97	18.9	10.76	9.13	3673.89	4286.74	9.13	4396.03	21632.45	17621.07	17730.13	30444.26	26195.71	25981.82	26430.57	21314.63	22765.09	22928.19	18122.94	22374.94	25218.89	26664.72	19845.72	25064.3	1.7666660885883936	0.00037596016108426203	2.7351115371846366	1.4515996669710634	up
MW0159668	HCO3-	HCO3	59.9856	60.9926	0.6009	9.7594	MS2-Insilico	71-52-3	HMDB00595	C00288	--	--	--	--	rp	257.89	193.64	121.03	174.49	309.28	241.87	243.29	44.65	146704.54	28789.54	67189.61	8552.42	163433.31	118119.98	50637.72	128718.1	79778.86	98425.22	125205.04	133063.01	45336.17	100981.3	131118.66	164174.77	1.765757024695546	2.438918855627368e-05	5.29617998415883	2.404952151193692	up
